# Peritoneal Lymphomatosis in a Pediatric Patient: A Peruvian Case Report

**DOI:** 10.7759/cureus.62750

**Published:** 2024-06-20

**Authors:** Junior Principe-Collazos, Anthony Ramos-Yataco, Natalia Nombera-Aznaran, Elizabeth J Ramos-Orosco, Lucero Sangster-Carrasco

**Affiliations:** 1 Department of Pediatrics, Edgardo Rebagliati Martins National Hospital, Lima, PER; 2 Department of Internal Medicine, University of Miami - Jackson Health System, Miami, USA; 3 Department of Internal Medicine, School of Medicine, Universidad Peruana Cayetano Heredia, Lima, PER; 4 Department of Pediatrics, School of Medicine, Universidad Peruana de Ciencias Aplicadas, Lima, PER

**Keywords:** unexplained ascites, peru, pediatric, peritoneal, lymphomatosis

## Abstract

While NHL commonly affects lymph nodes, peritoneal lymphomatosis causing ascites is rare in pediatric patients. We present a unique case of DLBC lymphoma in a Peruvian child presenting as ascites associated with peritoneal lymphomatosis. The 11-year-old boy was admitted with ascites and dyspnea. Physical examination revealed collateral circulation, abdominal distension, and diminished breath sounds. Investigations led to a suspected diagnosis of peritoneal tuberculosis; however, a laparoscopic biopsy showed granulomatous infiltration consistent with high-grade diffuse B-cell lymphoma. The peritoneal lymphomatosis causing ascites is uncommon, and its initial presentation as peritoneal symptoms is even rarer. Differential diagnosis between peritoneal tuberculosis and DLBCL involvement can be challenging due to both shared signs and symptoms. Staging systems, such as the International Pediatric NHL Staging System, aid in determining the extent of the disease. DLBCL has a good prognosis, with treatment regimens such as the LMB-89 protocol showing high overall survival rates. Awareness of DLBCL's atypical presentations is crucial for timely diagnosis and management in the pediatric population. To conclude, children with ascites represent a diagnostic challenge posed by overlapping symptoms with other conditions, such as tuberculosis, and the need for a comprehensive approach to rule out different etiologies. Additionally, it is important the prompt treatment to avoid complications.

## Introduction

Lymphomas are a type of cancer that arises from cells such as lymphocytes and histiocytes in different locations of the reticuloendothelial system. They are classified into two main types: non-Hodgkin lymphomas (NHL) and Hodgkin lymphomas (HL). Additionally, lymphomas are the third most common childhood malignancy, and NHL accounts for approximately 7% of cancers in children younger than 20 years in high-income countries [1,2]. In Peru, between 2015 and 2019, the National Institute of Child Health reported 43 cases of lymphomas in children, including 23 NHL cases. Of these cases, only one female preschooler was diagnosed with DCBL [3,4]. NHL can be classified into nodal and extranodal lymphomas that are characterized by multiple painless nodes. Cervical lymph nodes, tonsils, and abdominal lymph nodes are commonly affected and the symptoms depend on the location of the affected area, such as dysphagia, sore throat, or asymptomatic enlargement of one tonsil [1]. Peritoneal lymphomatosis causing ascites is hardly ever reported in the pediatric population. Here, we present a unique case of diffuse large B-cell lymphoma (DLBCL) presenting as ascites associated with peritoneal lymphomatosis in a Peruvian child.

## Case presentation

An 11-year-old boy with abdominal distension was brought to our Emergency Department (ED). He had a previous diagnosis of ascites, based on an abdominal ultrasound. On admission, heart rate was 98 beats per minute, respiratory rate was 38 breaths per minute, blood pressure was 110/60 mmHg, and oxygen saturation was 98% on room air. On review of systems, the patient complained of dyspnea and weight loss, and fever was denied. On physical examination, collateral circulation and abdominal distension with shifting dullness were noted, along with diminished breath sounds in the left lower base, and no lymphadenopathies were noted. Paracentesis and thoracentesis were performed (Table 1) due to possible spontaneous bacterial peritonitis; the patient was started on cefepime, metronidazole, and vancomycin.

**Table 1:** Laboratory Results

	Normal range	Results on admission
Serum Escherichia coli	Absent	Positive
Ascitic Pseudomonas fluorescens/putida	Absent	Positive
Ascitic fluid color	Clear	Yellow
Ascitic fluid aspect	Clear	Cloudy
Ascitic fluid coagulum	Absent	Absent
Ascitic fluid glucose (mg/dL)	70-100	25
Ascitic fluid protein (g/dL)	<4.1	3.4
Ascitic fluid leukocytes (cells/mm^3^)	<300	5004
Ascitic fluid bacteria	Absent	Gram negative
Serum ascites albumin gradient S.A.A.G	<1.1	0.6
Ascitic fluid Mycobacterium tuberculosis	Absent	Negative
Ascitic fluid malignant cells	Absent	Negative
Prothrombin time (sec)	10.50-13	13.90
International normalised ratio	0.85-1.15	1.17
Activated partial thromboplastin time(sec)	25.0-37.0	31.40
Thrombin time (sec)	16.0-21.0	16.1
Fibrinogen (mg/dL)	200-400	340.40
Hemoglobin (g/dL)	13.5-17.5	10.2
Hematocrit (%)	41-53	31.6
Mean corpuscular volumen (fL)	80-100	74.2
Mean concentration hemoglobin (pg/cell)	25.4-34.6	23.9
Mean corpuscular hemoglobin concentration (Hb/cell)	31-36	32.3
Platelets (10*3 cells/mm^3^)	150-400	922
White blood cells (cells/mm^3^)	4,000-11,000	12,930
Neutrophils (%)	54-62	66.9
Monocytes (%)	3-7	11.8
Lymphocytes (%)	25-33	20.6
Eosinophils (%)	1-3	0.5
Basophils (%)	0-1	0.2

During the second day of hospitalization, the patient complained of edema in the right lower limb. A Doppler ultrasound revealed a deep venous thrombosis. Anticoagulation was started, and the patient was transferred to the pediatric intensive care unit (PICU) for further monitoring. Upon admission to the PICU, his heart rate was elevated (126 per minute), his respiratory rate was elevated (28 breaths per minute), his blood pressure was 104/64 mmHg, and his oxygen saturation was 97% on room air.

**Figure 1:**
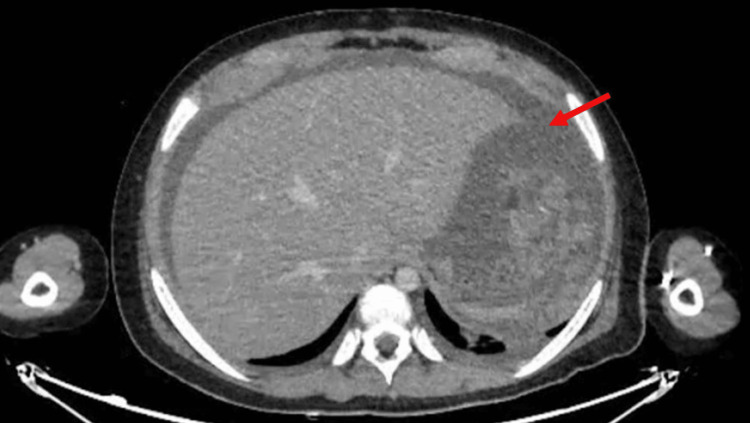
Abdominal Computed Tomography Red arrow indicating ascitic fluid

**Figure 2:**
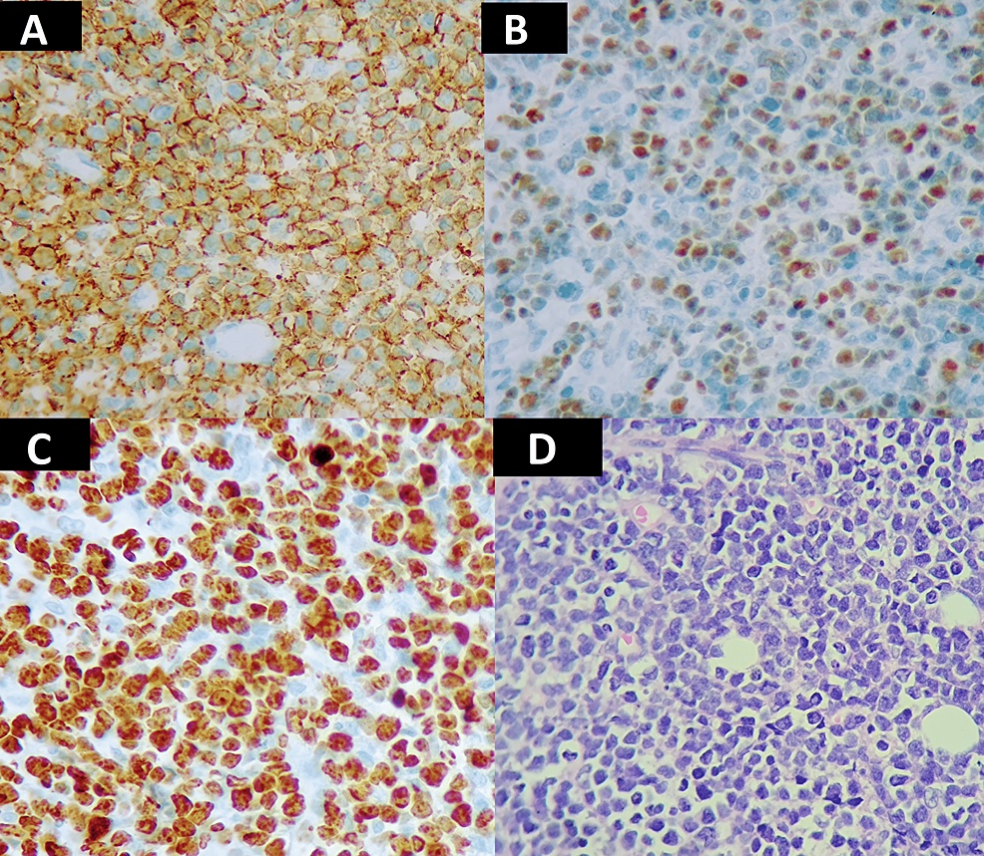
Histopathological Examination of the Peritoneal Biopsy A) The histologic examination of the laparotomy biopsy specimen shows the proliferation of lymphoid atypical cells with hematoxylin and eosin staining. B) CD20 positive. C) BCL-6-positive. D) Ki67 proliferation index > 95%

Abdominal computed tomography (CT) revealed ascites and peritoneal thickening (Figure 1). After analyzing the peritoneal fluid (Table 1) and conducting imaging tests, the healthcare team arrived at a potential diagnosis of peritoneal tuberculosis (TB), which is particularly prevalent in Peru (a country with a high incidence of extrapulmonary TB), even though the patient received the Bacillus Calmette-Guérin (BCG) vaccine, and there was not an epidemiological history of TB. In multidisciplinary rounds, the decision to perform a laparoscopic biopsy of the peritoneum was taken. During surgery, a thickened and friable peritoneum with diffuse granulomatous infiltration - without caseous necrosis - of cerebroid consistency was found, and multiple peritoneal biopsies were taken. Furthermore, the polymerase chain reaction for TB was negative. Histopathological and immunophenotypic findings were CD20 and BCL-6-positive, consistent with high-grade diffuse B lymphoma (Figure 2). Subsequently, the patient was started on chemotherapy treatment. The child had a good prognosis. He was discharged 48 days after arriving at the hospital.

## Discussion

Lymphomas (HL and NHL) are the third most common childhood malignancy, and NHL accounts for approximately 7% of cancers in children younger than 20 years in high-income countries [1]. High-grade lymphomas of B-cell origin are the predominant type of NHL observed in children, with Burkitt lymphoma being the most frequently diagnosed subtype [5]. DLBCL is a relatively uncommon type of NHL in children and adolescents, accounting for approximately 10%-20% of cases in this population [6]. It is rare before the age of four, but its incidence increases with age and it is more commonly diagnosed in males, with a male-to-female ratio of 2:1 [7]. In Peru, we have a few studies about lymphomas. The National Institute of Child Health reported 43 cases of lymphomas in children during 2015-2019. Only one female preschooler was diagnosed with DLBCL [4].

DLBCL in children often manifests as localized disease with focal lesions found in organs such as the liver, spleen, or lungs or a mediastinal mass [8]. Extensive infiltration of the peritoneum secondary to lymphomas is rare, and it is even more uncommon for it to initially manifest as peritoneal symptoms [9]. In a case series of patients with peritoneal, mental, and mesenteric lymphoma involvement, the most frequent subgroup of NHL was DLBCL; ascites was found in 75% of the patients [10]. In this case, the peritoneum was primarily involved, causing ascites and spontaneous bacterial peritonitis. As a result, it is noteworthy for being one rare case that documents this particular combination of symptoms.

Based on the serum ascites albumin gradient (SAAG), our patient had a possible exudate with increased polymorphonuclear neutrophils (PMN) and cell count, which correlates with possible bacterial or TB infection and malignancies [11]. The diagnosis of primary peritoneal lymphoma involvement may be challenging. TB remains a worldwide health concern, especially in low-income countries, such as Peru. It causes morbidity and mortality, especially in neonates and children with risk factors such as oncological problems and immunosuppression, among other diseases. According to the WHO, in 2018, one million children under 15 years old had TB; approximately 25% of whom died of the disease [12]. In Peru, there is no exact information about epidemiology, but the incidence of TB is on average 30 children affected per 100 million children under 15 years. Moreover, it has a high risk of spreading [13]. Peritoneal TB, even though rare, can mimic the signs and symptoms of an NHL such as ascites [14]. Furthermore, peritoneal thickening, which was found in our patient's abdominal CT, may be present in both TB and DLBCL. The SAAG in our patient was also consistent with an exudate due to increased PMN and cell count, which correlates with possible bacterial or TB infection and malignancies [15]. Based on a possible bacterial spontaneous peritonitis, our patient was put on a broad-spectrum antibiotic with further peritoneal fluid analysis (Xpert PCR) to rule out TB. Although Xpert PCR peritoneal fluid analysis allows a rapid diagnosis, its low sensitivity may require confirmatory tests such as histopathology. An antituberculous drug trial could be considered in tuberculosis-endemic areas [6]. In this case, the Xpert PCR test came out negative, ruling out the diagnosis. Subsequently, a peritoneal biopsy confirmed DLBCL lymphoma.

Oncological conditions are known to increase the occurrence of venous thromboembolism (VTE), which is a significant contributor to both morbidity and mortality. VTE incidence in lymphoma depends on cancer type, stage, and therapy. Studies have shown that NHL is associated with a higher incidence of VTE (6.5%) as compared to HL (4.7%) [16]. Additionally, high-grade NHL raises VTE risk over low-grade NHL. Patients with DLBCL have a particularly high frequency of symptomatic VTE episodes (12.8%), with 37% at diagnosis, reducing median survival [17,18].

The Saint Jude Staging System was the first staging in the NHL and is still in use today. However, it only considers the location of the disease, bone marrow, and central nervous system involvement (CNS) [19]. Nowadays, children with NHL are subclassified into different stages, ranging from low-risk to high-risk, according to the Berlin-Frankfurt-Munster Group Report [6]. In 2015, the American Society of Clinical Oncology updated the international pediatric NHL staging system (IPNHLSS), which incorporates new histological patterns, extranodal dissemination, advanced imaging technology, and new diagnostic methods such as monoclonal antibodies, PCR, immunohistochemistry, flow cytometry, cytogenetics, and fluorescence in situ hybridization (FISH) analysis [9]. In our patient, malignant ascites are due to NHL with peritoneum involvement. Furthermore, the Ki- 67 proliferation index was 95%, and the immunochemistry test was positive for CD20 and BCL-6. According to the IPNHLSS criteria, this is consistent with stage III DLBCL.

DLBCL is a treatable condition with a good prognosis depending on the stage of the disease. Woojung Jeon et al reported a single-center experience in the pediatric population with a diagnosis of NHL. Out of 85 patients with a diagnosis of NHL, 29 (35.4%) had DLBCL. Among these patients, all of them received the LMB-89 protocol (cyclophosphamide, vincristine, doxorubicin, prednisone). Regarding the prognosis, two patients (6.89 %) had stage I DLBCL with a 100% five-year overall survival rate, 13 patients( 44.8%) had stage II DLBCL with a 100% five-year overall survival rate, 11 patients (37.9 %) had stage III DLBCL with a 90.9% five-year overall survival rate, and three patients( 10.3%) had stage IV DLBCL with a 66.7% five-year overall survival rate [10]. Our patient had a diagnosis of stage III DLBCL and also started chemotherapy treatment with the LMB-89 protocol. The response was adequate; currently, he has no more complications.

## Conclusions

To conclude, it is important to recognize that DLBCL can manifest as peritoneal lymphomatosis, leading to complications including ascites. This case provides a unique perspective, showcasing the diagnostic challenges posed by overlapping symptoms with other conditions, such as tuberculosis, and the need for a comprehensive approach to rule out different etiologies. Additionally, novel diagnostics and treatment approaches, such as the IPNHLSS criteria and LMB-89 protocol, offer promising prospects for better outcomes. Clinicians should be aware of this atypical presentation to mitigate mortality and morbidity in the pediatric population diagnosed with DLBCL.

## References

[1] Singh R, Shaik S, Negi BS, Rajguru JP, Patil PB, Parihar AS, Sharma U (2020). Non-Hodgkin's lymphoma: a review. J Family Med Prim Care.

[2] Woessmann W, Quintanilla-Martinez L (2019). Rare mature B-cell lymphomas in children and adolescents. Hematol Oncol.

[3] (2024). Cancer stat facts: NHL - diffuse large B-cell lymphoma (DLBCL). https://seer.cancer.gov/statfacts/html/dlbcl.html.

[4] Gálvez C, Mendoza M, Espíritu N, Carrillo E (2021). Clinical, epidemiological and pathological characteristics of lymphomas in patients from the National Institute of Child Health of Breña - Peru, 2015-2019. An Fac Med.

[5] Minard-Colin V, Brugières L, Reiter A (2015). Non-Hodgkin lymphoma in children and adolescents: progress through effective collaboration, current knowledge, and challenges ahead. J Clin Oncol.

[6] Salzburg J, Burkhardt B, Zimmermann M (2007). Prevalence, clinical pattern, and outcome of CNS involvement in childhood and adolescent non-Hodgkin's lymphoma differ by non-Hodgkin's lymphoma subtype: a Berlin-Frankfurt-Munster Group Report. J Clin Oncol.

[7] Reiter A, Klapper W (2008). Recent advances in the understanding and management of diffuse large B-cell lymphoma in children. Br J Haematol.

[8] Mande R, Roy Moulik N, Shet T (2022). Clinicopathologic profile and treatment outcomes of children with diffuse large B-cell lymphomas: experience from a tertiary cancer Center in India. J Pediatr Hematol Oncol.

[9] Rosolen A, Perkins SL, Pinkerton CR (2015). Revised International Pediatric non-Hodgkin lymphoma staging system. J Clin Oncol.

[10] Jeon W, Koh YK, Kang S, Kim H, Koh KN, Im HJ (2022). Clinical characteristics and treatment outcomes of children and adolescents with aggressive mature B-cell lymphoma: a single-center analysis. Blood Res.

[11] Alshuwaykh O, Cheung A, Goel A (2022). Clinical characteristics and outcomes in those with primary extrahepatic malignancy and malignant ascites. BMC Gastroenterol.

[12] (2024). WHO: Tuberculosis. https://www.who.int/es/news-room/fact-sheets/detail/tuberculosis.

[13] Thomas TA (2019). Tuberculosis in children. Thorac Surg Clin.

[14] Vaz AM, Peixe B, Ornelas R, Guerreiro H (2017). Peritoneal tuberculosis as a cause of ascites in a patient with cirrhosis. BMJ Case Rep.

[15] Shahed FH, Mamun-Al-Mahtab Mamun-Al-Mahtab, Rahman S (2016). The evaluation of serum ascites albumin gradient and portal hypertensive changes in cirrhotic patients with ascites. Euroasian J Hepatogastroenterol.

[16] Komrokji RS, Uppal NP, Khorana AA, Lyman GH, Kaplan KL, Fisher RI, Francis CW (2006). Venous thromboembolism in patients with diffuse large B-cell lymphoma. Leuk Lymphoma.

[17] Soares M, Caruso P, Silva E (2010). Characteristics and outcomes of patients with cancer requiring admission to intensive care units: a prospective multicenter study. Crit Care Med.

[18] Ahmed OF, Kakamad FH, Salih AM, Mustafa MQ, Mohammed SH (2020). Non-Hodgkin’s Lymphoma presenting as deep venous thrombosis; A case report with literature review. Int J Surg Case Rep.

[19] Murphy SB (1980). Classification, staging and end results of treatment of childhood non-Hodgkin's lymphomas: dissimilarities from lymphomas in adults. Semin Oncol.

